# Exploring barriers to switching “on time” to second-line antiretroviral therapy among nurses in primary health care facilities, Ekurhuleni Health District, South Africa

**DOI:** 10.1371/journal.pone.0284996

**Published:** 2023-04-26

**Authors:** Immaculate Sabelile Tenza, Christine Njuguna, Pumla Pamella Sodo, Aviva Ruch, Joel Msafiri Francis, Olufemi Babatunde Omole, Richard Cooke, Samuel Agbo, Laurel Baldwin-Ragaven

**Affiliations:** 1 Faculty of Health Sciences, South African Research Chairs Initiative (SARChI), School of Public Health, University of the Witwatersrand, Johannesburg, South Africa; 2 Faculty of Health Sciences, School of Nursing Science, North-West University, Potchefstroom, South Africa; 3 Faculty of Health Sciences, Department of Family Medicine and Primary Care, School of Clinical Medicine, University of the Witwatersrand, Johannesburg, South Africa; Nigerian Institute of Medical Research, NIGERIA

## Abstract

**Background:**

Ensuring that all HIV-infected people receive antiretroviral therapy (ART) and achieve viral suppression are key South African strategies to end the AIDS epidemic in the country. National HIV treatment guidelines recommend an immediate switch to second-line ART following virological failure with first-line ART. Nurses based in district health facilities are at the forefront of implementing this recommendation. While there are often delays in switching and in some instances no switch, the reasons for and barriers to delayed switching are not well understood at the primary care level.

**Aim:**

To explore the views of frontline nursing staff about factors contributing to delayed switching of patients who have failed first-line ART regimen in Ekurhuleni district, South Africa.

**Methods:**

A qualitative study was conducted among 21 purposively sampled nurses who provide HIV treatment and care to patients in 12 primary health care (PHC) facilities in Ekurhuleni Health District, Gauteng Province, South Africa. Individual in-depth interviews explored nurses’ experiences regarding their recognition of virological failure and understanding of “on time” switching to second-line ART. Interviews probed the circumstances contributing to delays in switching. After digital audio recording and transcription, manual inductive thematic analysis was used to analyse the data.

**Findings:**

Multiple barriers were identified: 1) Healthcare provider factors included a lack of knowledge and confidence coupled with demotivation in the workplace; 2) Patient issues similarly comprised a lack of knowledge as well as resistance to being switched to another drug regimen and loss to follow up; 3) Systems factors were poor facility leadership, shortages of medication, staffing constraints, and the inability to trace laboratory results, especially for migrant patients.

**Conclusion:**

Reasons for delayed switching of patients to second-line ART are multifactorial and require integrated interventions at health provider, patient and health system levels.

## Introduction

Globally, an estimated 38.4 million people were living with HIV (PLHIV) in 2021 [1]. Of these number, 25.8 million were from Africa, accounting for 68% of the global HIV prevalence [1]. South Africa had an estimated 7.5 million PLHIV in 2021, yielding an adult (15–49 years) HIV prevalence of 18.3% [2]. South Africa also has the largest antiretroviral (ART) programme globally with just over 5 million people on ART [3].

Efforts in South Africa to strengthen the diagnosis, treatment and management of HIV and AIDS have emphasized building human resource capacity at primary care facility level for delivering ART. These efforts included the development of ART guidelines which are aligned with those of the World Health Organization [4], involving non-governmental organizations to support treatment delivery and training of PHC nurses on nurse-initiated management of antiretroviral treatment (NIMART) [5].

At the time when this study was conducted in April 2021, the first line regimen for treatment of adult patients in the South African ART guidelines consisted of a fixed-dose combination tablet consisting of two nucleoside reverse transcriptase inhibitors (NRTIs). Typically, this regimen included Tenofovir and Emtricitabine, or Tenofovir and Lamivudine, plus an integrase inhibitor such as Dolutegravir or a non-nucleoside reverse transcriptase inhibitor (NNRTI) such as Efavirenz or Nevirapine for women of child-bearing potential who are not on reliable contraception or pregnant women [5]. Following initiation of first line therapy, the South Africa ART guidelines recommend viral load (VL) monitoring at six [6] months, 12 months and yearly after, if viral suppression has been achieved [6]. For patients with persistently elevated VLs, however, enhanced adherence counselling should be provided together with the need to exclude co-existing opportunistic infections, drug-drug interactions and incorrect dosing [6]. A follow up VL should be repeated three months after this intervention; and, if the result is still ≥1000 copies/ml, this establishes virological failure. Thus, two consecutive elevated VLs (HIV RNA ≥1000 copies/ml) on two separate occasions at least three months apart for patients on Efavirenz or Nevirapine-based ART regimens, or at least two elevated VLs (≥1000 copies/ml) over two years on a dolutegravir-based ART regimen [6], should be recognized as a “medical emergency” requiring immediate action. The recommendation therefore is an immediate switch to a second-line ART regimen at the same visit when VL is recognized [6]. Second-line ART regimens in South Africa typically comprise two NRTIs and a boosted protease inhibitor (lopinavir/ritonavir or atazanavir-ritonavir) or an integrase inhibitor such as dolutegravir [6].

Although South African guidelines recommend immediate switching of patients to second-line ART following virologic failure, there are often delays in initiating such second-line regimens; and, in some instances switching does not occur at all [7].

A meta-analysis of adult HIV cohorts from Sub-Saharan Africa (SSA) reported a low incidence rate of switching to second-line of 2.65 per 100 person years, since treatment failure rates are estimated at 5–10% [8]. Other studies from SSA have also reported sub-optimal switching in adult HIV cohorts after virologic failure, with proportions of those eligible for switching who actually receive second-line ART ranging from 39% to 66% [9–13]. Time to switching to second-line ART after confirmed virologic failure is also not immediate, with delays in most HIV cohorts in SSA ranging from 3.8–16.7 months [9–13].

South African cohort studies have reported similar results, with estimated proportions of adult ART patients eligible to switch to second-line ART ranging from 21.6%-43.5% [14–16]; and, the median time to switch to second-line ART after confirmed virologic failure being prolonged by five to six months [16, 17]. A recent multi-cohort study conducted in 52 South African urban and rural primary health care clinics reported the length of time to switch to second-line ART between 48–96 weeks, with an average of three non-suppressed VL laboratory results before being switched successfully [15]. South African studies have also highlighted predictors for switching to second-line ART [14] as well as identified patient and health system factors associated with delayed switching [17, 18]. For example, being male [16] and being of older age at ART initiation [15] were demographic factors associated with delayed switching to second-line ART. Similarly, having higher CD4 counts at the start of ART [14, 15] and lower viral load at the time of ART failure [14] were risks for delayed switching. Poor health professional adherence to virologic monitoring guidelines, missing VL laboratory results in patients’ files [18] and incomplete prescribers’ notes were also associated with delays in switching patients to second-line ART [17, 18].

Evidence points to how delaying or failing to respond to virologic failure negatively impacts treatment outcomes, including an increased risk of mortality [5, 6], treatment resistance [7] and the inability to achieve virologic suppression even after initiating second-line ART [8, 9]. Not switching timeously to second-line ART also threatens the UNAIDS target of reducing global inequalities to treatment access and efficacy [19]. In the South African context, healthcare professionals at the PHC level, particularly nurses, are at the forefront of HIV management and attend to the clinical needs of most PLHIV. In Ekurhuleni, a metropolitan district in the urban Gauteng province, reports suggest that other outcomes of HIV management such as retention in care and loss to follow up are largely sub-optimal and that only a minority (25%) of HIV-positive patients are even initiated on ART at CD4 counts >500 cells/ mm^3^ [20]. Additionally, anecdotal evidence suggests that there are often delays in switching patients to second line ART. However, there has not been any empirical studies conducted to unveil the reasons for these delays. More importantly, there is little understanding of the contextual experiences of nurse clinicians at PHC level, more so, that PHC nurses are well placed to first recognize treatment failure and then independently initiate second-line ART or refer complex patients to doctors for regimen change. Therefore, gaining an understanding of the lived experiences of these nurses is crucial for developing strategies to improve clinical practice at the PHC frontlines, the first port of call for most patients on ART. The aim of this study was to explore the barriers to timely switching of patients who have failed first-line ART, from the perspective of nurses who manage HIV management at primary care level.

## Methods

### Study setting

This study was conducted in the Ekurhuleni Health District, one of five districts in Gauteng Province (GP), situated to the east of Johannesburg and extending to the border of Mpumalanga Province. The district is divided into three geographical sub-districts namely: North Sub-district, South Sub-district and East Sub-District. According to Statistics South Africa, Ekurhuleni had nearly four million inhabitants in 2021, with an average household income of R29,400 per year and an unemployment rate of 31% [21]; and, over half a million children (513,912) received social grants [22]. The HIV prevalence in Ekurhuleni was 15.4% in 2017 [23]. All primary healthcare facilities in Ekurhuleni offer integrated services, from which a patient may receive HIV and other disease-based health care from a single clinician, most frequently a nurse. Patient headcounts are <5000 per month at the municipally-run primary care clinics which receive visits from sessional generalist doctors 1–3 times per week; whereas, in the larger provincially administered (CHCs), headcounts are >5000 per month and there is a specialist family physician on site to support nursing services [22].

### Study sites and participants

All primary care nurses who had at least six months’ work experience of providing adult HIV management in district health facilities were eligible to participate. Facilities were selected based on the differential monthly number of adult HIV visits between the CHCs and the PHC clinics were conveniently selected one provincial CHC and three municipal PHC clinics from each of the three sub-districts in Ekurhuleni, resulting in a total of 12 facilities from which the sample was drawn. All nurses in the 12 facilities meeting the eligibility criteria above were invited to participate in the study. Those who responded were purposively recruited to ensure diversity in the range of professional nursing categories, years of HIV treatment experience and history of HIV/ NIMART training, and to ensure representation from the selected facilities.

### Data collection tool

A semi-structured interview schedule was developed for data collection. It included questions on the management of patients who have failed first-line ART and probed factors influencing delays in switching to second-line ART. Interviews explored provider, patient, and health system barriers to timely switching. After review for content validity and clarity by researchers, the interview schedule was piloted with three nurses from one of Ekurhuleni primary care facilities which was not included in this study. No changes were made following the pilot.

### Data collection

A trained male field worker with experience in conducting surveys and in-depth interviews contacted the facility managers of the 12 selected PHC facilities to arrange a site visit, in order to introduce the study and recruit participants. After nurses agreed to participate, return visits were organized for interviews as per their time preferences. One on one interviews were conducted and digitally recorded by the field worker between April–June 2021. All participants who agreed to participate completed the consent forms and demographic questionnaire. Questions guided by the interview schedule were posed to participants. All interviews were conducted in English and lasted between 60–90 minutes each. Data was collected until saturation was reached. This occurred at interview number #19; however, two additional interviews were conducted to ensure saturation.

### Ethical considerations

Ethical approval to conduct the study was obtained from the University of the Witwatersrand Human Research Ethics Committee (HREC) Medical, certificate #M2008100. Permission to access the facilities and recruit staff was received from the Ekurhuleni District Research Committee. During the recruitment process, it was made clear to nursing staff that participation was entirely voluntary and would have no impact on their employment situation. On the day of the interview, the fieldworker obtained written informed consent from respondents for both participation in the study and for audio-recording. Interviews were conducted in a room with a closed door in the facility to ensure privacy. To safeguard participants’ anonymity, codes were used instead of participant and facility names.

### Data analysis

Digital recordings of the interviews were professionally transcribed verbatim. Following transcription, one of the researchers (IST) listened to all of the audio recordings while reading the transcripts to ensure completeness of the data. A team of four researchers (IST, PS, HM, and AR) performed data analysis using a line by line coding for the first four transcripts, each from a different facility to ensure methodological rigour [24]. The team members coded independently and made notes on their reflections from the data. Subsequently, the team developed, interrogated, and evaluated themes for similarities and differences in meaning. Several meetings were held to discuss codes, themes, and emerging sub-themes. The remaining transcripts were analysed in the same manner, with several meetings held to reach inter-coder agreement. Sub-themes and related quotes were shared electronically with the entire research team; and meetings were held to discuss themes and sub-themes until agreement was reached.

### Trustworthiness

We applied Lincoln and Guba’s criteria of trustworthiness to examine rigour in this study [25]. We listened repetitively to the audio-recorded interviews to ensure credibility of the findings and read and reread the transcripts to ensure prolonged engagement with the data. Four researchers coded the data, and inter-coder agreement was reached to ensure dependability. We ensured confirmability by attaching the extracts of narratives in the transcripts to support the emerged themes.

## Findings

A total of 21 nurses participated in the study. Table 1 presents the participants’ characteristics:

**Table 1:** Nurse participant characteristics.

	N (%)
**Age**	
** 20–39**	9 (43)
** 40–50**	10 (48)
** >60**	2 (10)
**Gender**	
** Male**	4 (19)
** Female**	17 (81)
**HIV management experience**	
** 6–12 months**	3 (14)
** 1–2 years**	5 (24)
** 2–5 years**	4 (19)
** > 5 years**	9 (43)

Table 2 presents the findings on what participants perceived as contributors to delayed switching despite first-line ART failure. The emerged themes are presented in terms of the categories of health care provider, patient, and health system-related factors.

**Table 2:** Factors contributing delayed switching.

Categories	Themes	Sub-themes
**Healthcare provider factors**		
	Lack of knowledge and confidence in switching	• Inadequate training • No interest to do NIMART training • Lack of confidence in ability to switch • Lack of motivation to read and follow guidelines • Poor reference to previous clinical notes
	Sub-standard consultation practices	
		• Too many adherence counselling sessions required • Nurses’ negative attitudes towards switching to second-line ART • Unhealthy nurse-patient relationships • Perceived lack of power or permission to switch
**Patient factors**		
	Lack of knowledge and negative attitudes to their ART treatment Unhealthy nurse- patient relationships Lost to follow up/transience	• Resistance to be switched
**Health system factors**		
	Leadership and management Lack of resources/constraints Inadequate systems to track/monitor laboratory results	• Over-reliance on non-governmental organizations (NGOs) to lead HIV treatment programmes • Lack of accountability • Staff shortages • Shortage of medications/medication stock outs

### 1. Healthcare provider factors

The healthcare provider factors included nurses’ lack of knowledge, demotivation, and lack of confidence, including sub-optimal standards of consultation practices.

#### Nurses’ lack of knowledge and confidence, including demotivation

The participants reported that non- or poor attendance at in-house training sessions may lead to insufficient knowledge and lack of confidence among nursing staff, resulting in avoidance of providing HIV care and delays in switching of patients to the second-line ART:

*“You know when I say there is lack of knowledge, our supporting NGO does provide with training but they (nurses) don’t want to attend. Like I am a NIMART nurse and they will ask who want to do NIMART, they will say I am tired, or I am doing this and that so they don’t want, but if I am not here they don’t initiate, they will wait for a NIMART nurse but if they are offered training, they don’t like (to attend)” [**DVE-NURS-03**]*

When probed for the reasons for lack of confidence respondents added that it was due to fear of the unknown and a need for refresher training:

*“Lack of confidence maybe, fear of the unknown” [**MMC-NURS-01**]*

*“Others are afraid to switch, they will say they are not confident enough. Somebody will say I am not exposed much, others will say I need refresher course, I have done, NIMART 15, 10 years ago” [**OLF-NURS-02**]*

The lack of knowledge did not only affect confidence but also judgement, attitude and nurses’ decisions leading to reluctance to switch. For example, one professional nurse shared her belief that there was no need to switch patients to second-line as they should be able to be maintained on first line ART.

*“You know what, I don’t understand this switching to the second-line because the goal is to get everybody to be on the first line, it (is) not like when we (are) switching the person to the second-line, he is going to be better. The second-line is wrong because when we switching the person to the second-line, it means that the person very soon can go to the 3rd regimen” [**TSK-NURS-02**]*

Nurses also complained that although they have the South African ART guidelines available at their workplace, these documents were lengthy and hard to understand. Hence, this resulted in a general lack of motivation to read the guidelines, in addition to their busy schedules:

*“There is really no time to read those thick documents*, *while on duty*, *when you see the document*, *you just loose interest to read it*, *there should be a way to simplify the guidelines*, *make them more user friendly*” [***MMC-NURS-01***]

#### Sub-standard consultation practices

Under this theme, participants reported that healthcare providers do not refer to previous clinical notes, and that numerous adherence counselling sessions, negative nurses’ attitudes towards switching, bad nurse-patient relationships and their perceived lack of power to switch, were contributory factors to delayed switching to second-line ART.

#### Poor reference to previous clinical notes

Lack of reference to previous clinical notes was underscored as a major contributor to delays in switching on time:

*“Let’s say this patient comes today and then I see that the patient might be in virological failure, and then l do counselling and all, maybe on the next visit, that patient is supposed to take the second viral load, but the next clinician will miss that viral load.” [**CYP-NURS-01**]*

*“I may see a patient today and quickly pick up that the viral load (laughing) is not going anywhere and then I would plan: okay we first do adherence counselling and then the next visit we do this and then the patient comes next week they don’t find me, they find another clinicians so you find that the other clinician don’t follow up on the previous management, the next clinician will even miss the previous management so such things most of the time are the things that will hinder the patient to be switched to the second-line”. [**JAB-NURS-01**]*

In such cases, the person who gets to see the patient on another occasion will have to initiate the switching to second-line, depending on the thoroughness of his/her consultation.

*“The first initiative is to prepare the patient to be switched because it won’t help me going through the file and checking on who saw the patient, what and what, so I just have to take the first initiative and prepare this patient for switching” [**JAB-NURS-01**]*

#### Numerous adherence counselling sessions

One of the other reasons for delaying switching on time was described as the nurses’ good intention to allow a patient to master adherence to the first line ART. In these circumstances, the nurses would provide numerous chances for counselling sessions to improve adherence. In some instances, they would continue to assess and observe the patients on first line ART, as they were concerned that failure to adhere to first line treatment would lead to a similar behaviour on second-line ART. This is what one of the nurses had to say:

*“We educate, educate, if that person is still using traditional healers, if that person is still not using condom(s), we got to educate about that. Like you cannot encourage somebody who is struggling with 1 tablet to move to the 6, like it’s not practical.” [**TSK-NURS-02**]*

#### Nurses’ attitudes towards switching to second-line ART

Participants believed one of the challenges leading to delayed switching is nurses’ attitudes towards switching patients to second-line ART. For example, one of the participants reported that switching to second-line ART should be happening at hospital level only, because being switched to second-line ART reflects poorly on a PHC facility when so many patients do not achieve virologic suppression on first line ART treatment. Hence, nurses would rather continue with adherence counselling.

*“At primary health care level, you cannot encourage patients to be on secondary ART because very soon when you are on secondary line ART. It means you are getting out of the clinic now because very soon you will be at the hospital. We don’t want patients to be on second-line” [**TSK-NURS-02**]*

#### Nurse-patient relationships

One nurse reported that poor interaction between the nurse and the patient discourages adherence to the treatment.

*“I think the other reason is attitude. I will call attitude out because some of our colleagues, the manner of approach of speaking to our patients, the patient would maybe take it as an offense or will take a decision, and say I am no longer going to that particular facility because of that particular nurse or particular nurse treated me this way, (I) am not going to take these pills just because of the manner of the approach I have been approached” [**KAT-NURS-01**]*

#### Perceived lack of power, authority or permission to switch

Most nurses believed that it was not within their scope of practice to switch patients to second-line ART. In such circumstances, they would have to refer patients to the medical doctor; and, if there were no doctor on site, they would have to refer patients to the hospital.

*“Because when you switch the patient from the first line to the second-line you need to refer the patient to the doctor, it is the doctor who initiate(s)”. [**RON-NURS-01**]*

*“We used to switch the patients to second-line, but we have been put on hold for now. If the patient must be switched to second-line, we must refer to the facility doctor.” [**CYP-NURS-01**]*

*“If we don’t have the doctor, and there is no one with NIMART and then we refer that patient to the hospital.” [**OLF-NURS-02**]*

The participants pointed out that in instances where a hospital referral was done, not all patients honoured their appointments, sometimes due to cost of transportation to the referred hospital, compounding delays in timeous switching.

### 2. Patient factors

Nurses’ perceptions of patient related factors contributing to delays included a lack of patient knowledge regarding the importance of switching to second-line ART treatment on time, resistance to being switched, including being dishonest and untraceable.

#### Patient’s lack of knowledge and poor attitude

Patient’s lack of knowledge on the importance of switching results in negative attitudes to being switched to second-line ART, and hence resistance to being changed from a single pill regimen to many pills.

“*They complain about taking these many pills because the one that they are used to is not so many but (now that it) is not working*, *we have to switch them to these many pills*, *they struggle to understand the benefits or decision to switch*” [***CYP-NURS-01***]

Participants also reported that some patients would be dishonest and move from one facility to another to pretend they are starting on treatment for the first time.

*“Patients will also not be truthful of what treatment they are taking” [**KAT-NURS-01**]*

*“In my experience it is very difficult to sort of maintain patients if they are going and shopping around (to different facilities) and not being consistent to one place. That is basically what I have experienced as leading to delays” [**KAT-NURS-01**]*

#### Resistance to being switched

Lack of knowledge also makes it difficult to accept being switched to second-line ART. Patient resistance included complaints about being switched to multiple tablets instead of a combination single pill, having to take doses more than once a day and the size of the pills.

*“They are running away from this double dose.” [JAB-NURS-01]*

*“Yeah, they do complain because especially the ones that we switch them from TEE which is one drug, to AZT, 3TC and Aluvia. So, if the client, if the client is now switched to AZT, 3TC and Aluvia, they are going to take 6 pills and they are used to 1 drug. So, sometimes it becomes like too much for them, the pill burden, so now they have to take 6 pills and they are used to 1 pill, so sometimes they do.” [**CYP-NURS-01**]*

*“Maybe the patient can say let me go and do what is right, maybe can you give me some time, maybe I can do right, but you can see that this viral load (means that it) needs to be switched, maybe the viral load is not suppressed, the patient needs to be switched, but the patients will keep saying that I need more time, I don’t want to go to the second-line, I don’t want to take more pills than I was taking one pill, I need more time, so it can also delay” [**RON-NURS-01**]*

#### Loss to follow up

One of the contributors to delays in switching patients to second-line was that some patients were difficult to trace, especially if they decided not to honour their scheduled clinic appointments and/or did not provide truthful contact details.

*“Another challenge is, yeah, sometimes the patients don’t come to their scheduled appointments, they miss their dates. But if they miss the date we call, we trace the patient but only to find out (that) maybe they gave us the wrong number, wrong address for tracing.” [**CYP-NURS-01**]*

*“They are those who (are) lost to follow maybe the patient moved to other province maybe, yes, the other challenge is that maybe the home tracing you find that the patient doesn’t belong to that address that can be the other challenge, maybe the phone is not working” [**RON-NURS-01**]*

### 3. Health system factors

Systemic factors, such as the failure of the facility manager to enforce on-time switching, the lack of resources at primary care level and the challenges of tracing laboratory results, were the critical issues raised as contributors to delays in switching patients to second-line ART.

#### Leadership and management

Some participants believed that there is lack of clarity regarding the facility manager’s role on supporting timeous switching. For example, one informant mentioned that she had never seen her facility manager being involved in monitoring HIV management, since this role had been seemingly shifted to the NGO supporting her facility.

*“We have an NGO dealing with the HIV, STIs, TB, uhmm. Yeah so, they are the ones that are leading the programme, I personally have never seen my manager monitoring delays in switching patients.” [**DVE-NURS-02**]*

#### Lack of personal accountability

Participants also lamented on the lack of accountability as part of performance management in cases where switching had not happened on time.

*“I haven’t seen anything being done when delayed switching is noted, except for in-service training but like yeah am thinking (of) accountability for not switching, I haven’t seen that” [JAB-NURS-01]*

One participant added that switching on time is not measured or prioritised in the same way as initiation on ART.

*“The manager doesn’t concentrate on switching patients to the second-line, their focus is on initiation of ART” [MMC-NURS-01]*

#### Lack of resources: shortages of staff and medication stock outs

*Staff shortages compromising quality of care*. Staff shortages often led to duties such as switching patients to second-line ART not being prioritized due to high patient-nurse ratios, contributing to delayed switching.

*“Another serious problem we had, there is shortage of staff, we have nurses and doctors that are dedicated, but unfortunately we are overwhelmed as well with the number of nurse-patient ratio. You find that for you to provide excellent service for a patient, you need to at least spent 30 minutes with this patient, especially a child, but because I guess the high nurse to patient (sic) ratio, you find that you can only see a patient for 5 minutes, and then you find that per day instead of seeing 25 you see something like 89, 90 patients. So, there is no quality, it is just quantity”. [**CYP-NURS-02**]*

*“No time for adherence counselling, because I mean before you switch, these people have to be counselled, so because of our queue, if we don’t have the counsellors, we normally do have but if we don’t have, they are gone for some meetings, you have to sit with the patients and counsel you know, there is no time” [**MMC-NURS-02**]*

#### Shortages of medications

Drug shortages were often due to delays in procurement processes, and the fact that second-line ART are scheduled drugs requiring special prescription by the doctor in certain instances. Participants lamented that in one instance they had to wait for 3–6 months for the necessary second-line drugs to become available.

*“(The) biggest problem that we have is shortage of medication ……. now that we have to start patients on TLD, there is no TLD, and we have already switched some, when they come, we don’t know what to do. Not only TLD but with other drugs as well. So that is a serious problem for us because it makes us look like we don’t know what we are doing. Another challenge is, sometimes the dolutegravir is sometimes out of stock, yeah, that’s with this dolutegravir, especially client(s) with renal impairment we have to switch them from TEE based to let’s say Abacavir 3TC and DTG, so, sometimes the dolutegravir is out of stock” [**CYP-NURS-01**]*

Other participants similarly pointed to patients’ lack of trust as a consequence of medication shortages and stock outs, as well as leading to forced defaults and delayed switching.

*“Patients lose trust in us because we are the first people they come to for help and then if we don’t have stock, we don’t have stock. At the end of the day, patients say what is the use of me going to the clinic while you don’t have adequate amount of stock? Yeah, then we lose that patient” [**KAT-NURS-01**]*

*“Other patient(s) will default, to say really and truly, they are saying these drugs are available, and now they are not available” [**MMC-NURS-02**]*

#### Inadequate laboratory tracking systems

Participants were of the opinion that the lack of a system to track a patient’s laboratory results was a contributor to delays in switching on time. This participant pointed to lack of a centralised record system which allows tracing progress of care if a patient has visited a different clinic and then returned to his/ her usual facility.

*“Patients lie too much. So we don’t have this [centralised record system] where we check if they are taking treatment in another facility, so if you use lab tracer, which started 4 years ago. So, if you use it, maybe the patient took medication 5 years ago, defaulted and come back, we will write on the file what happened but he will come back as a new patient because we don’t have anything else to check”. [**DVE-NURS-03**]*

The same challenge becomes a particular concern for providing appropriate follow up care to cross-border migrant patients.

*“No, we are failing, we are failing because the lab tracker is for South Africa. Zimbabwe, we don’t, Mozambique, and so on. We don’t have records of immigrants who are on ART” [**DVE-NURS-03**]*

*“If the patient verbalise(s) that I am taking treatment from Zimbabwe, we will consider that when planning treatment, but if he said he is a new patient while he is dishonest, we will take him as a new patient. Well, nothing will show, (as) they don’t appear on lab-tracker. So, it is going to be like that, it will be a new a patient” [**DVE-NURS-03**]*

## Discussion

This study sought to investigate provider, patient and health system barriers that lead to delayed switching of patients following viral load suppression failure to a second-line ART regimen in Ekurhuleni Health District in South Africa. We found that health care provider-related barriers contributing to delayed switching included: sub-standard clinical practices as well as a lack of knowledge, confidence and motivation. None of our study participants pointed to years of experience as a barrier contributing to delayed switching. Patient-related barriers included poor patient attitudes to switching and loss to follow-up. Health system-related barriers included poor leadership/ management, lack of resources and inadequate systems to track or monitor laboratory results.

Nurses’ lack of knowledge, self-confidence and perception of authority as barriers to timely switching were attributed to a number of issues such as a lack of interest in attending training, lack of motivation to read clinical guidelines, and in some instances insufficient training opportunities to improve and upgrade knowledge and skills. In other settings, demotivation to read and apply general clinical guidelines by nurses has been attributed to such documents being “non-user-friendly” and difficult to understand [26, 27]. Likewise, a qualitative study from South Africa reported nurses’ perceptions of guidelines as too complex; and, this is a major barrier to clinicians’ adherence to HIV and tuberculosis guidelines [28]. The development of less cumbersome, electronic, or pocket handbook guidelines may therefore be a health system imperative to improve HIV care in district health settings in South Africa. This highlights the need to empower nurses with knowledge in form of continuous professional development to enhance their knowledge on ART switching. Furthermore, there is a need to ensure that all nurses are NIMART trained so that they have confidence and power to prescribe or switch the patients to second level of ART.

Lack of interest in attending training as well as insufficient training opportunities might have contributed to the knowledge gaps and low confidence levels reported by nurses in our study. Lack of knowledge of HIV management has negative consequences such as poor quality of care [29]. Tackling knowledge gaps in HIV care amongst nurses may require strong leadership skills that will facilitate the prioritization of continuous professional development and in-service training. Crowley et al. (2021), in a narrative review of enablers and barriers of nurse-initiated antiretroviral treatment in South Africa, reported training of nurses as an important enabler [30]. Similarly, Makhado et al. (2020) reported continuous education (in-service, refresher, follow-up training) as facilitating HIV guideline adherence by nurses in South Africa. Low confidence and perceived lack of authority in switching patients was a barrier contributing to delayed switching, and may be mitigated by ongoing mentorship and supervision, which are evidence-based strategies that can improve confidence of nurses and the quality of HIV care [28, 31].

Sub-standard consultation practices were reported as another health care provider barrier to switching to second-line ART. This included the failure to review previous clinical notes as well as negative nurses’ attitudes towards switching patients to second-line ART. Omission of review of previous clinical notes falls short of the standard for good patient care, impinges on continuity of care and is of major concern. One regional South African study found that insufficient or scanty prescriber’s notes as well as the inadequate review of clinical notes in a patient’s file by a subsequent health care provider contributed to delayed switching of adult patients to second-line ART [17]. This gap could be addressed by regular audits on the quality of patient records and reflective feedback incorporating standards of professionalism that together would build a culture of accountability among clinicians, both for the processes and outcomes of care they provide.

Resistance to switching to second-line ART was reflected in the numerous and unnecessary counselling sessions, poor nurses’ attitudes to switching and perceived lack of power or permission to switch. There were reported misconceptions that switching must occur at hospital level or only when authorized by a medical doctor. In 2008, World Health Organization provided guidelines for task shifting to improve access to care for PLHIV [32]. South Africa adopted these guidelines through HIV care decentralization from hospital to PHC level, task shifting HIV care from doctors to nurses through the nurse-initiated management of ART (NIMART) programme. This strategy has improved access to HIV care and led to positive patient outcomes in PLHIV [33–35]. Yet, despite the evidence base for task-shifting, our findings highlight a remaining disconnect between policy and current practice and underscores the importance of role clarity and standardization of practices across the district. These barriers can be mitigated by clear communication on roles in switching and enforcement of such roles.

The health needs of PLHIV are diverse and not limited to only medical care. Meeting these needs requires a coordinated and whole-person approach that should involve multi-disciplinary teams [28].

Loss to follow-up was reported as a patient barrier to timely switching to second-line ART. This has clinical implications noting that data from studies conducted in low-middle income countries, including South Africa, suggest that loss to follow-up often occurs between the time when the second elevated viral load is taken and the next clinical visit [18]. Consequently, clinicians are unable to switch patients to second-line ART in a timely manner. Ekurhuleni, just like any other health district in Gauteng has a significant migrant population that may negatively affect on-going retention in HIV treatment care. Our findings therefore highlight the need to strengthen the electronic health records and tracing system, through cross verification of a patient’s personal details to enhance traceability of patients, minimize loss to follow up, and thereby improve timely switching of ART regimens when necessary.

Negative patient attitudes to switching to second-line ART primarily due to pill burden concerns and lack of continuity of care due to switching health care providers were reported as barriers. This finding suggests the importance of considering patients’ preferences and respect for patient agency and autonomy when implementing guidelines. Furthermore, evidence from a previous study reported that when patient autonomy was considered through inclusion of patients in their own treatment plans, this facilitated nurses’ adherence to HIV and TB guidelines [28].

Lack of resources, in particular second-regiment ART drugs and staff shortages, were reported as health system-related factors that contributed to delayed switching. A previous study reported ART shortages as a reason for patients disengaging from HIV care and becoming lost to follow-up [36]. While drug stock outs are not unique to ARTs and within Ekurhuleni, the consequences are clinically dire (as in other national health priority programs) [37]. These include avoidable morbidity and mortality, since patients who are lost to follow up are likely to present sicker at a later stage with reduced survival. Health systems managers therefore need to strengthen drug supply chain protocols by health facilities and ensure proper communication with pharmacy colleagues when stocks are running low. Complementary monitoring and oversight by civil society advocacy groups may further ensure adequate ART stock levels by ensuring transparency and agitating against stockouts [37, 38].

Staff shortages were cited as a barrier to timely switching, mainly due to nurses’ pressure to attend to more patients rather than spend the necessary time to explain and ensure adherence to the a required second-line ART switch: “quantity over quality”. Staff shortages have been reported as a barrier to timely switching in a qualitative study that measured barriers to adherence to HIV and TB guidelines amongst nurses [28]. To address this, health system managers need to prioritize the implementation at primary care level of the South African Human Resources for Health policy norms and standards [39], in addition to filling vacant nursing posts and addressing retention concerns, particularly of younger nurses who are attracted to posts in the private sector or overseas.

In this study, we found poor leadership and the lack of accountability for poor clinical practices as health system barriers to timely switching. This was mainly attributed to inadequate supervision of nurses by facility managers, as well as a lack of accountability by clinic managers due to the over-reliance on NGO partners to run the HIV programme at district level. A similar lack of supervision has been reported to hamper adherence to clinical guidelines by nurses in other studies [27, 28]. The lack of visible leadership in some of the facilities highlights a need for capacity building to strengthen facility manager’s role in HIV management and improve the cooperation between the health care teams and their NGO partners.

Lastly, we found that there are inadequate health information systems to track or monitor laboratory results (elevated viral loads), particularly amongst highly mobile patients. This highlights the limitations of the current electronic laboratory monitoring systems that do not integrate with health systems elsewhere. More integration of health information systems is therefore crucial in addressing the health care needs of mobile populations on ART.

Our study has some limitations. We only included perceptions and experiences of primary care nurses and did not capture those of patients, other clinicians, and health system administrators, which is an area for future research. Context is important for the interpretation of qualitative studies and the views of participants may change depending on setting and time. As this study explored the views of nurses, there is the potential for social desirability and information bias. The study findings being from a qualitative enquiry may also not be generalizable. However, to the extent that efforts were made to ensure credibility and trustworthiness of findings, they may be transferable to other settings depending on similarity of contexts.

Notwithstanding the above, most studies on delayed regimen switching in HIV programs are of quantitative design; and, to our knowledge, this is one of the few that provide a qualitative perspective on this issue, particularly from nurses’ perspectives. The study further contributes lessons on the need to strengthen the training of nurses on clinical guidelines, clinician-patient relationships and attitudes and scope of practice. It also calls for improving human resource and drug management, and the need for clinical oversight and governance embedded into the HIV program in South African at district level. The utility and effectiveness of a pocket-sized or electronic summary in providing easy and real-time access to the latest HIV management guidelines, and consequently promoting timely switching, are areas that future operational research could investigate. Likewise, the possible solutions addressing these barriers have been proposed; however, it is another area for future research to evaluate which solutions/recommendations are effective as part of implementation science.

## Conclusion

Multiple nurse, patient and health system-related factors influence the timely switching of first-line ART regimen patients with virological failure to second-line ART regimen in a typical South African health district. Delayed switching is hinged on knowledge and “soft” skills gaps, sub-standard clinical practices by nurses, ambiguity of roles, poor nurse-patient relationships, weak management, leadership gaps and systems failures. All of these call for ongoing training, monitoring and clinical governance to ensure timely switching to second-line ARV regimens at PHC level.

## List of abbreviations

AIDS: Acquired immunodeficiency syndrome
ART: Antiretroviral therapy
HIV: Human immunodeficiency virus
NIMART: Nurse initiated managed antiretroviral therapy
NGO: Non-governmental Organization
NRTIS: Nucleoside reverse transcriptase inhibitors
PLVHIV: People living with HIV
